# Use of chest radiography screening for TB: a re-evaluation of the Kolín study

**DOI:** 10.5588/ijtld.22.0216

**Published:** 2022-10-01

**Authors:** A. Schwalb, J. C. Emery, R. M. G. J. Houben

**Affiliations:** 1 TB Modelling Group, TB Centre, London School of Hygiene & Tropical Medicine, London, UK; 2 Department of Infectious Disease Epidemiology, London School of Hygiene & Tropical Medicine, London, UK; 3 Instituto de Medicina Tropical Alexander von Humboldt, Universidad Peruana Cayetano Heredia, Lima, Peru

Dear Editor, Current TB care and prevention policies have resulted in a slow (<2%) annual decline in disease incidence,^1^ meaning we are not on track to reach the 2030 Sustainable Development Goals.^2^ Historically, mass chest radiography (CXR) screening programmes were widely used, in part because of their sensitivity for pulmonary TB.^3^ However, following a 1974 WHO Expert Committee report, mass CXR screening was mostly abandoned in the past half-century. It was concluded that mass CXR screening had “no significant effect on the occurrence of subsequent smear-positive cases” and, given the resource requirements, was not a cost-effective tool to interrupt transmission.^4^ One of the key sources cited in support of this viewpoint are the results from a carefully conducted long-term study on mass CXR screening and TB epidemiology in the district of Kolín, Czechoslovakia, from 1960 to 1972.^5^–^7^

However, because costs for mass CXR screening are rapidly dropping (due to technological advances, such as digitalisation and computer-aided detection of pulmonary abnormalities), we felt it timely to reevaluate the Kolín study; specifically, whether or not the idea that mass CXR screening does not add epidemiological value still holds up. We re-examined the data from two key publications to address the questions: 1) whether or not there was a decline in TB incidence during the study period, and 2) if there was a decline, to what extent was this due to mass CXR screening. Before the study, the TB burden in Czechoslovakia was high.^6^ Kolín District housed approximately 100,000 inhabitants across rural and urban areas, with an annual TB incidence of 151.8 per 100,000 in 1955–1959.^6^ The study’s objective was to observe epidemiological trends in a region covered by a TB programme over the 12-year study period, which included, among other measures, systematic Bacillus Calmette-Guérin vaccination and treatment, follow-up of cases and repeated mass CXR surveys of the population aged of ≥15 years.^6^,^7^ During the study period, five mass CXR surveys were conducted to obtain several point-prevalence estimates. CXR films were independently and blindly evaluated by two physicians and stored for comparison in subsequent surveys.^6^

To address our first question, we extracted data from a plot of the period prevalence of bacillary pulmonary TB, defined as the number of notified cases (through study procedures) within a calendar year, for the whole study period (Krivinka et al.,^7^ p 64). The graph used a logarithmic scale (replicated in the Figure, left), which visually underestimates the decline of total and new cases.^7^ However, plotting the data on a linear scale and fitting a linear regression curve to the logarithm of the cases by the least-squares approach (Figure, right), we see that the total number of cases declined annually by 13.6% (95% CI 9.4–17.9). It is worth mentioning that TB prevalence increased noticeably in 1961 due to the first mass examination of the study; further peaks also correspond to years when mass CXR screening took place. The final year of the curve in 1972 also corresponds to a mass screening year, which implies that 1973 would have seen a further drop, mirroring previous patterns. A slightly lower decrease in the number of new cases was observed (annual decline of 10.8%, 95% CI 3.9–18.0). Although the study mainly attributed the overall decline to a drop in prevalence due to the effective treatment of chronic patients (annual decline of 42.8%, 95% CI 37.7–48.2), a substantial reduction in new TB cases was also observed over the whole period. The decline in TB burden also coincided with a period of national economic growth, impacting the socio-economic status of the study population.^8^ It is useful to note that the annual decline differed between the two study periods, with a steeper decline in total cases occurring in 1960–1964 (annual decline of 17.2%, 95% CI 9.3–51.3) compared to 1965–1972 (10.6%, 95% CI 1.1–20.9), which suggests that interventions in the study had the largest impact shortly after implementation. Although less striking, it still exceeds the current annual global decline in TB five-fold.^1^ Thus, either impact would be welcomed by most, if not all, TB programmes today.

**Figure:**
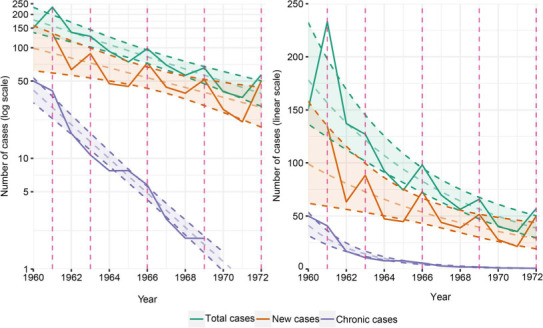
Bacillary pulmonary TB cases in Kolín, Czechoslovakia, 1960–1972. Number of bacillary pulmonary TB cases detected in Kolín, Czechoslovakia, from 1960 to 1972, categorised as total, new (newly discovered cases in a calendar year) and chronic (continuous bacillary excretion for 2 years) cases, with corresponding regression lines. The original figure from Krivinka et al. is shown on the left (log scale), and the adapted figure is shown on the right (linear scale).^7^ Dotted vertical lines indicate the year when a mass CXR screening took place. CXR = chest X-ray.

The second question is the extent to which the decline of TB experienced in Kolín was due to mass CXR screening. Ascribing causality is challenging, but it is important to work through. Ideally, CXR screening would identify new cases early, during what we now refer to as the subclinical phase (i.e., before systems develop). Furthermore, the smear status of new cases is more likely to be smear-negative (i.e., have contributed less to transmission).^9^ For the period 1960–1964, mass CXR contributed to the detection of 61% (148/241) of new TB cases, of which (16%, 23/148) were smear-positive (see Appendix Table 10 in Stýblo K, et al.^6^). For the period 1965–1972, the contribution to the detection of new cases declined to 38% (102/270); however, a larger proportion were smear-positive (29%, 30/102; see Table 2 in Krivinka R, et al.^7^). These numbers were interpreted to show that CXR was ineffective at detecting smear-positive cases. However, it is worth highlighting that even in that period, symptom-based passive case-finding contributed to 48% (129/270) of new cases, of which 43% (56/129) were smear-positive cases. One could therefore argue that the effectiveness of CXR screening for detecting new smear-positive cases was not substantially inferior, and nor was it irrelevant for the observed decline. In contrast, the conclusions surrounding the “impracticality” of CXR were fixed on its lack of detection of smear-positive cases. However, this focus prioritised interrupting rather than preventing intensive transmission and therefore warrants closer scrutiny.

As defined in a chapter in *Toman’s Tuberculosis*, case detection is the early detection of individuals discharging and transmitting tubercle bacilli that is “carried out in order to treat the sources of infection so as to alleviate their suffering and to render them non-infectious”.^5^ Based on this description, CXR perfored well by primarily identifying early cases, most of which are less infectious (non-smear-positive), and providing them with timely treatment to prevent future suffering. Nevertheless, in the same chapter Toman considers the Kolín study an example against the effectiveness of mass CXR screening. We would argue that identifying smear-negative, asymptomatic cases could, and maybe should, be regarded as a benefit instead of a limitation.

Aside from the Kolín study, mass CXR screening has shown promise as an effective measure in other settings. In Cape Town, South Africa (1950–1970), a temporary decrease in TB notification rates coincided with population-wide active case-finding using miniature CXR.^10^ Since then, the increased availability and reduced cost of CXR have allowed for its wider use, especially in regions with precarious healthcare systems.^3^ It may be particularly valuable in settings with a high TB and HIV burden, as CXR is a cost-effective tool for TB screening in HIV-positive individuals.^11^ Additionally, complementary use of molecular testing for TB further reduces requirements on infrastructure and costs.^12^

As the TB community looks for new solutions for the persistent global TB problem, we should revisit our beliefs about what historical data can tell us. Although mass CXR screening has challenges, we show here that its historical performance was better than originally perceived.
